# Supplementary material for: Predation and fragmentation portrayed in the statistical structure of prey time series
Source: BMC Ecol. 2009 May 6;9:10. doi: 10.1186/1472-6785-9-10 (PMC2689204; doi:10.1186/1472-6785-9-10)
Supplement: Additional file 2 — Voles and related classes ODDox Documentation. ODDox documentation of the agent-based model (ALMaSS) applied by Hendrichsen et al. The documentation is started by activating main.html. [file 1472-6785-9-10-S2.zip › Vole_ODDox/main.html]

ALMaSS ODDox: Voles and related classes ODDox Documentation

- Main Page
- Related Pages
- Classes
- Files

# Voles and related classes ODDox Documentation

### 1.01

# Chris J. Topping Department of Wildlife Ecology National Environmental Research Institute, University of Aarhus Grenaavej 14 DK-8410 Roende Denmark 22nd January 2009

  
  


---

  

### A note about ODDox

This model description follows broadly the ODD protocol for describing individual- and agent-based models (Grimm et al. 2006) and consists of three main elements. The first provides an (O)verview, the second explains general concepts underlying the models (D)esign, the last, (D)etail, provides the information about the various model constructs in detail. The Detail section presented has been implemented by using the Doxygen program (http://www.doxygen.org)on the commented source code, creating a html-based documentation. In this way the source can be viewed and navigated through in an efficient and manageable manner. A major advantage is that connections between objects, methods and functions are hyperlinked and can therefore be followed easily together with the source code, even without more than a very basic understanding of programming.  
  
In addtion to the change of Detail to using Doxygen, the section originally entitled 'Submodels' in ODD has been alterned to 'Interconnections'. The reason for this is that submodels will be described under the classes that are covered in the Doxygen part of the ODDox and that classes with connectsions to other classes described are not submodels but entities existing at the same hierarchical level. The section 'Interconnections' is therefore used to highlight important linkages to the main classes described in any one section of the ODDox. Note also that the ODDox for ALMaSS consists of several individual documentation sections. Interconnections allows the reader to navigate to those descriptions that are of primary relevance.  
  
A note about code structure: This program was written in C++ code which is an object-oriented language. Objects (e.g. a male hare) are termed 'instances' of a 'class' (e.g. Hare\_Male). Classes are arranged in a hierarchy whereby information contained in a base class is available to descendent classes. For an example see the class diagram at the top of TALMaSSObject. This diagram shows how TAnimal is descended from TALMaSSObject, and subsequently THare is descended from TAnimal, and Hare\_Male from THare. Understanding this basic concept and terminology will be an advantage when traversing through the code using the hyperlinks since methods (functions, behaviours) and attributes that are not modified in the descendent class need to be viewed in the base class.  
This method of programming reduces code size and eases code development. An example of this approach can be seen by comparing Hare\_Juvenile::st\_Dispersal with Hare\_Female::st\_Dispersal. In this case the female dispersal uses the juvenile code, then adds special female behaviour afterwards.  
  


---

### Overview

**i *Purpose***    

The field vole model was developed as part of the ALMaSS framework (Animal Landscape and Man Simulation System) which was primarily constructed as an assessment tool for use in answering management and policy questions related primarily to changes in farm management, landscape structure and land use. The ALMaSS system was designed to be as flexible as possible since the precise applications of the system were not known in advance. The field vole model is therefore designed to be a flexible model capable of translating changes in it?s simulated environment into patterns of changes in population sizes, reproductive outputs and distribution with time. Flexibility is a function of the high level of detail implemented in the model with the aim of simulating Microtus agrestis responses as realistically as possible, whilst still maintaining a manageable code volume and parameter space.  

**ii *State* *variables* *and* *scales***   

ii.1 Low level variables that characterize the low-level entities of the model  
  
ii.1.a Individuals  
  
The simulated vole population consists of individual voles that shared a set of common state variables and some that were unique to each life-history class.  
  
The state variables common for the vole classes:   
1) Vole\_Base::m\_Age - The age of the object in days.  
2) TAnimal::m\_Location\_x - The x coordinate in the landscape where the individual were born.  
3) TAnimal::m\_Location\_y - The y coordinate in the landscape where the individual were born.  
4) Vole\_Base::IDNo - Identification number associated with the object.  
5) Vole\_Base::m\_Sex - Male or female.  
6) Vole\_Base::m\_Mature - Boolean variable indicating whether the vole has reached maturity (==true).  
7) Vole\_Base::m\_TerrRange - The radius of their territory.  
8) Vole\_Base::m\_LifeSpan - Maximum number of days left for the vole to live if not subject to other mortality.  
9) Vole\_Base::m\_Weight - Weight of the vole in grams.  
10) Vole\_Base::m\_StarvationDays - Number of days the vole has been starving.  
11) Vole\_Base::m\_DispVector - The current dispersal direction.  
12) Vole\_Base::m\_Have\_Territory - Boolean variable set to true if they have a territory.  
13) Vole\_Base::m\_Reserves - The objects reserves and thereby the number of days they can survive without food.  
14) Vole\_Base::CurrentVState - Behavioural state of the object.  
15) Vole\_Base::m\_pesticideInfluenced - For specific pesticide use only.  
16) Vole\_Base::m\_pesticideInfluenced2 - For specific pesticide use only.  
  
Unique state variables for the females:  
1) Vole\_Female::m\_NoOfYoung - Number of young in the nest.  
2) Vole\_Female::m\_Pregnant - Boolean variable set at true if the female is pregnant.  
3) Vole\_Female::m\_DaysUntilBirth - Number of days until the female gives brith.  
4) Vole\_Female::m\_YoungAge - The age of the young produced.  
5) Vole\_Female::m\_BornLastYear - Receives the value of 1 if she was born the previous year.  
  
Unique state variables for the male:  
1) Vole\_Male::m\_fertile - Indicates the fertility status of the male. Default is fertile == true. Used primarily in pesticide applications.  
  
Administrative state variables used to speed of facilitate operation of code:  
1) Vole\_Base::m\_OurPopulation - Pointer to the population manager. This is needed by all individuals to access population manager functions.  
2) Vole\_Base::SimW - Provides the individuals with the width of the landscape.  
3) Vole\_Base::SimH - Provides the individuals with the height of the landscape.  
  
ii.1.b Habitats  
  
ALMaSS is spatially explicit and has a square raster GIS based representation of a landscape with a default resolution of 1 m^2^. The size of the landscape can change with the scenario simulations and can be in the scale of e.g. 10 km x 10 km; 5 km x 5 km or 1 km x 1 km to make the model flexible and applicable to a wide variety of scenarios and data availability. The length of a simulation run is flexible as well and can be set to any number of years with the time-step set at one day as a default. This resolution and extent make it feasible to model vole behaviour at a fine scale whilst still keeping run-times within sensible bounds.  
  
State variables used by the vole model and associated with each habitat unit were:   
1) m\_polyrefs - Identification number associated with each unique polygon.   
2) m\_elems - Land-cover code for each polygon.   
3) m\_farms - If the code for the land-cover were a field then a farm owner was associated with it and carried out farm management on the owned units.   
4) m\_vege\_type - Vegetation type for the polygon (if any vegetation grows in the polygon. 5)  
  
The landscape model in ALMaSS was weather dependent and the state variables associated with the weather sub-model were:   
1) m\_temptoday - Mean temperature on the given day (Celsius).   
2) m\_windtoday - Mean wind speed on the given day (m/sec).   
3) m\_raintoday - Total precipitation on the given day (cm).  
  
The ALMaSS Landscape component is responsible for handling all landscape, weather and management related sub-models. These include 74 management plans for Danish crops, crop and habitat related vegetation and insect growth models, and miscellaneous human management models. Documentation of these is outside the current scope of this documentation, but please refer to the following documentation: 13-17.  
  
  
**iii *Process* *Overview* *and* *Scheduling***    
  
The field vole model consists of three life-stages; juvenile, female and male separated into two classes of males and females. During a life-cycle the vole object transitions through a number of behavioural stages based on information obtained during its current behavioural stage and messages received from other objects in the simulation. This scheduling together with many other functions are contained in the Vole\_Population\_Manager class (see submodels). The voles enter the simulation at the location of the females nest and at 14 days of age (WeanedAge==13) (Innes and Millar, 1994; Leslie and Ranson, 1940) as either a female or male with m\_Mature == false. They will enter the state of Vole\_Female::st\_Evaluate\_n\_Explore (female) or Vole\_Male::st\_Eval\_n\_Explore (male) and start searching the area for a suitable territory.  
  
Every new day for a vole in the simulation starts off by evaluating and exploring (Vole\_Female::Step Vole\_Male::Step dependent on their sex). Other behaviours may ensue dependent upon the information gathered during this process. Possession of a territory is a prerequisite to breeding. Males have the possibility of breeding with any females with territories located totally or partially within the male?s territory. Voles that find themselves in older voles territories of the same sex with an overlap of >50% must move. The criteria for assessing territory quality vary with season, and for the male in the breeding season include the proximity of females.  
  
iii.a Scheduling   
  
The simulation time-step is one day, meaning that at the end of each day the state of the simulation is consistent with all objects having completed one day of activity. All farming and other human activities are assumed to occur at the beginning of the day on which they are scheduled, thus knowledge of these events is available for all animal models which are considered subsequently.  
  
A hierarchical approach to scheduling of animal objects is used to overcome the problems of currency between interacting objects discussed by Topping et al. (1999). Although the computer is operating serially, the objects must be scheduled such that they appear to operate in parallel. For the vole model and all other ALMaSS models this is achieved using a state machine to move objects between behavioural states and by dividing the time-step into three sections, namely BeginStep, Step and EndStep. BeginStep all objects execute their BeginStep code before any objects can execute Step code. All male objects are called in a random sequence and execute the step code, then all females also in a random sequence. When all male and female voles are finished with BeginStep this process is then carried out for Step. There is a significant difference between Step and BeginStep and EndStep. In Step a vole must signal that it is finished with Step behaviours for this day otherwise after the first pass through all individuals, a second pass is carried out for those individuals that have not indicated that the Step is done with. This process is repeated until all individual objects are finished with the Step code. At this point the EndStep is called in the same way as BeginStep.  
  
The BeginStep/Step/EndStep structure allows for a very flexible combination of interlinked behaviours (although only used to a limited extent by the vole), and permits efficient use of resources e.g. by removing dead voles in the BeginStep or EndStep before they waste CPU resources in Step. Handling of all scheduling via BeginStep/Step/EndStep is performed in the base class Population\_Manager.  
  
iii.b Vole States  
  

  
The figure above shows the overall structure and relationships for the female (A) & male (B) behavioural states. Details of the these states are provided below together with direct links to the program code to facilitate understanding of the way in which states and transitions are used.  
  
Evaluate and Explore   
Vole\_Male::st\_Eval\_n\_Explore   
Vole\_Female::st\_Evaluate\_n\_Explore  
If the vole does not possess a territory (m\_Have\_Territory == false) or if the habitat quality in the current territory falls below FHabQualThreshold1 (or MHabQualThreshold1) the vole will undergo dispersal (Vole\_Female::Dispersal and Vole\_Male::Dispersal). If habitat quality is (M/)FHabQualThreshold2 or above the vole will carry out an exploratory movement in one randomly picked direction with weight 3 (see Movement), to assess the habitat quality of a nearby area. If a new and better territory can be established there then the vole moves, otherwise the vole will return the old location. When the vole spends time in a habitat with a quality above (M/)FHabQualThreshold2 its body condition will improve by one per day to a maximum value of three. For the voles undergoing dispersal forced by low habitat quality, they will loose body condition and begin accumulate starvation days for each day spent in poor conditions. Accumulation of starvation days to 21 leads to death.  
Territory quality assessment is an important part of this behaviour. The size of the territory is linearly correlated to the weight of the vole and is dependent on its gender (see Table 1 & EndStep), and the quality is based on a mean quality of this area.   
For mature males: if it is the breeding season the territory will first be assessed for the presence of older voles. If the centre of their territory is located within an older vole?s territory or if more than 50% of the territory overlaps with other voles of the same sex, it is assumed unacceptable. If these criteria are fulfilled the male vole assesses the territory for the number of female voles located within it and optimizes the territory to entail as many females possible based on the boundaries of other males territory and the limitations of their own range of territory. If no females are present in the territory the male will not settle.  
For all other situations (and females): the habitat quality is assessed by scoring each 1m^2^ according to its quality as defined in Table 2. The habitat quality is defined as the total of these values divided by the number of voles with the centre of their territory overlapping with the assessed area. During the breeding season all voles check the extent of overlap of their territory with older voles of the same sex. This simulates a seasonally territorial system, where the territoriality breaks down outside the breeding season as described by Erlinge et al. (1990) and Agrell et al. (1996).  
Uses: Vole\_Base::AssessHabitat, Vole\_Base::CalculateCarryingCapacity, Vole\_Base::CalculateCarryingCapacity  
  
Infanticide  
Vole\_Male::st\_Infanticide   
If the male has moved beyond the bounds of his original territory and established in a new area he will attempt to kill all new born voles within his new territory. In this way he will increase his reproductive access to females in the new territory (Agrell, 1998). The probability of killing the young is dependent upon their age and to what extend the female defends her nest. Infanticide figures for the field vole have not been available hence values from the study of Heise and Lippke (1977) on the closely related common vole (Microtus arvalis) have been used (Table 1).  
  
Maturation  
Vole\_Male::st\_Maturation   
Vole\_Female::st\_BecomeReproductive   
A young vole will become mature when it attains the age of maturation during the breeding season (Table 1). When it is mature it is capable of mating once it has established a territory. Vole\_Male::st\_JuvenileExplore Ensures that the young male voles start to move away from the nest on the first day of independent life.  
  
Dying  
Vole\_Base::st\_Dying  
The vole can reach this stat by three routes 1) it may be killed by a predator; 2) by external factors e.g. farm operations or traffic; 3) or it may reach the end of its life span (Table 1). Farm management as an external mortality factor cause death to the voles by the disturbance of grazing life-stock (25%), harvest and cutting (50%) and soil cultivation (75%). In all cases only those voles present on the field under farm operation at the time the management was carried out were subjected to agricultural mortalities.  
  
Mating   
Vole\_Female::st\_Mating  
A female will mate if she mature during the breeding season and has established a territory. If the territory of a male is overlapping the females position she will be mated. If more than one males territory is overlapping her position she will be mated with the male closest to her location and gestation will start, which for the field vole last for 21 days (Leslie and Ranson, 1940; Innes and Millar, 1994). During mating a copy of the males genome is passed to the female and stored until used when any eventual offspring are weaned. Gestation Vole\_Female::st\_UpdateGestation Counts down the days of gestation and implements any reproductive impacts associated with pesticide accumulation (if any).   
  
Give Birth   
Vole\_Female::st\_GiveBirth   
The number of young is adjusted to the age of the mother and the month of the year they are delivered as observed by Andera (1981). Gestating females which have been over-wintering will produce a higher number of young than females reaching maturity within the same year as their birth (Table 1). The gender of the young is determined by random allocation based on the assumption of an even sex ratio (Myllyimaki, 1977). Young will only be produced by females with good body condition (assumed equivalent to three days spent in a territory with HQT above (M/)FHabQualThreshold2 (i.e. above marginal).  
  
Lacation   
Vole\_Female::st\_Lactating   
The female will enter this state when she has given birth. She will locate the best habitat within her territory to place her nest. She will stay within a radius of 6 m of the nest and feed her young (Jensen and Hansen, 2001). After lactation is over (i.e. the young are weaned) the female will do the special evaluation and explore as described in the section Special Exploration. The young produced at this point will carry a genome dependent upon a recombination of the male and female genes (the males genome was stored on mating). Recombination is controlled by void GeneticMaterial::Recombine and mutation can occur at this point depending upon the genetic settings used (outside the scope of this description).  
  
Special Exploration   
Vole\_Female::st\_Special\_Explore   
This behaviour is called after weaning of a litter when the female needs to expand her territory again. This does not use the Dispersal function so avoids extra mortality at this point and simulates the real phenomenon of sudden increase in daily home range after weaning (Agrell, 1995). In this case the exploratory movement is carried out in all eight directions rather than the daily single directional explore done in Exploration above.  
  
iii.c Important methods:  
  
BeginStep   
Vole\_Male::BeginStep   
Vole\_Female::BeginStep   
The BeginStep one of the three time-step divisions. This is called once for each vole before Step and EndStep. The main function here is to remove voles that die before they take up CPU resources in the Step code. It is also be used to check for pesticide accumulation levels in pesticide simulations.  
  
Step   
Vole\_Male::Step   
Vole\_Female::Step   
The Step is one of the three time-step divisions. This is called repeatedly after before Step and before EndStep, until all voles report that they are done with Step. Most of the behaviours are controlled by moving voles between behavioural states in Step (for other models this is also done in BeginStep and EndStep). When a vole is done for the day it will signal this by setting StepDone==true. NB that a call to one behaviour may trigger a call to another behaviour on the next call to step inside the same time-step. In this way a daily cycle of activity can be undertaken (e.g. do reproduction and explore in the female)  
  
EndStep   
Vole\_Male::EndStep   
Vole\_Female::EndStep   
The EndStep one of the three time-step divisions. This is called once for each vole after BeginStep and Step. The main function here is to remove voles that have died during step and otherwise to grow if not at max weight. Vole EndStep also checks if the vole was killed due to human management and determines the potential territory size. For newly adult voles the territory size will be set to the minimum size and grow larger until the vole has reached the maximum weight (Table 1). Male territory is generally larger than the females and a larger vole is capable of sustaining a larger territory (Agrell et al., 1996; Erlinge et al. 1990).  
  

  
Movement   
Vole\_Base::MoveTo   
Movement is an essential and difficult concept to model in an ABM. In ALMaSS, a standard method of implementing terrestrial movements was used and was defined by three parameters:  
1) A directional vector, indicating the preferred direction.   
2) A weight, indicating the strength of the bias towards the directional vector.   
3) The number of steps, indicating the maximum allowed distance per time step.   
  
Weights varied between zero (all eight directions allowed) and four (only the preferred direction allowed). For each step, habitat quality was evaluated in each of the possible directions and movement occurs to one of the suitable locations chosen at random. Different combinations of the movement variables resulted in a diverse set of potential animal-movement patterns across a landscape. Animals followed the above rules unless they became stuck, whereby the preferred direction and vectors would lead them into unsuitable habitats. In this case, the vector is altered by plus/minus one until movement is again possible. This basic movement model was enhanced by the addition of preferential choice between suitable habitats. Thus, the vole only makes a random decision if there is more than one location where the habitat quality is highest. This enhancement also requires a fourth parameter that describes the chance of an animal accepting a suitable, but not the most suitable habitat, to move to. This allows the animal to restrict its movement across some unsuitable habitats e.g., roads, but occasionally to cross them.   
  
The problem of voles getting stuck is avoided by preventing backwards movement, but allowing the vector to change by +/-1 if there is no legal choice of habitats to step onto given the current vector and weight.  
  
Uses: Vole\_Base::MoveQuality, Vole\_Base::MoveTo, Vole\_Base::DoWalking & Vole\_Base::DoWalkingCorrect   
  
  
  
Dispersal   
Vole\_Base::Escape   
As movement but with the weight of the directional vector set at three. In this way the vole is able to travel further away from the starting point than by entering the movement state.  
  

|  |  |  |
| --- | --- | --- |
| **Parameter Name** | **Value** | **Source** |
| Male Reproductive Start  VolePopulationManager.cpp::cfg\_GrassStartGrowth | 2 weeks after the grass starts to grow | Myllyimaki (1977); Jensen and Hansen (2001).   Simulated to be when there has been 14 days with a mean temperature above 4 degrees C |
| Male reproductive finish  Vole\_all.cpp::MaleReproductFinish | 1st October | Estimated from Erlinge et al. (1983) |
| Max male territory (radius m)  Vole\_all.cpp::MaxMaleTerrSize | 20 | Agrell et al. (1996) |
| Max female territory (radius m)  Vole\_all.cpp::MaxFemaleTerrSize | 16 | Erlinge et al. (1990) |
| Min male territory (radius m)\ Vole\_all.cpp::MaleTerritoryRange | 12 | Erlinge et al. (1990) |
| Min female territory (radius m)  Vole\_all.copp::FemaleTerritoryRange | 8 | Jensen and Hansen (2001) |
| Infanticide risk by age  Vole\_all.cpp::InfanticideChanceByAge | % mortality with 1-9 days old {97,86,75,64,54,43,32,21,11} | Heise and Lippke (1997) Microtus arvalis |
| No of young (depending on year of birth and the month)  Vole\_Population\_Manager::ReproTable | Female born this year {0,0,0,0,4,4,4,4,3,2,3,0}/n Female born last year {0,0,4,4,5,5,5,5,4,4,0,0} | Andera (1981) |
| Max female movement (m)  Vole\_all.cpp::FemaleMovement | 50 | Jensen and Hansen (2001) |
| Max male movement (m)   Vole\_all.cpp::MaleMovement | Max movement m with age in months{10,40,70,110} | Jensen and Hansen (2001) |
| Gestation period (days)   Vole\_all.cpp::TheGestationPeriod | 21 | Leslie and Ranson (1940), Innes and Millar (1994) |
| Weaned age (days)  Vole\_all.cpp::WeanedAge | >13 days old | Leslie and Ranson (1940), Innes and Millar (1994) |
| Weight at weaning (g)  Vole\_all.cpp::WeanedWeight | 5 | Myllyimaki (1977) |
| Max male weight (g)  Vole\_all.cpp::MaxWeightM | 60 | Myllyimaki (1977) |
| Max female weight (g)  Vole\_all.cpp::MaxWeightF | 55 | Myllyimaki (1977) |
| Growth per day (g)  see Vole\_Male::EndStep | Assumed linier growth, taking 90 days to reach maximum weight from weaning. | 90 days is estimated from Myllyimaki (1977) to be the time required during the growth period to attain max size. No growth was assumed to occur during over-wintering. The curve was linearized for speed of calculation. |
| Minimum male reproductive age (days)  Vole\_Male::st\_Maturation | 40 | Clarke (1977) |
| Minimum female reproductive age (days)  Vole\_all.cpp::MaturationChance | 20 | Clarke (1977) |
| Maximum life-span (days)  Vole\_Base::m\_LifeSpan | 14 to 20 months | Myllyimaki (1977) |
| Starvation days before death  Vole\_all.cpp::MaxStarvationDays | 21 | Estimation of 5 days based on some availability of food even in very poor habitats and a higher risk of predation for dispersing voles, Myllyimaki (1977). However, subsequent testing indicated that this value was too low and resulted in unrealistically low dispersal success. It was therefore increased to this arbitrary value. |
| Habitat quality threshold levels (HQT)   Vole\_all.cpp::MHabQualThreshold1  Vole\_all.cpp::MHabQualThreshold2  Vole\_all.cpp::MHabQualThreshold3 | 1.0, 1.5, 2.0 | Arbitrary value (see text description of Vole\_Male::st\_Eval\_n\_Explore and Vole\_Female::st\_Evaluate\_n\_Explore above). |

**Tabel 1. Overview of the parameters in the vole model with presented together with source information.**

|  |  |  |  |
| --- | --- | --- | --- |
| **Type** | **Vegetation Characteristics** | **Typical Landscape Elements** | **Habitat Score** |
| Optimal habitat | Cover > 80%, height > 40 cm | Ungrazed and uncut grassland, old set-aside, field and road margins | 3 |
| Sub-optimal habitat | Cover > 40%, height > 10 cm | Young tree plantations, cereal crops undersown with grass, dry meadow or heathland | 2 |
| Marginal habitat | Cover < 40% or height < 10 cm | Grazed or cut grassland, cereal and grass crops | 1 |
| No habitat | No grass | Roads, mature tree stands, other areas with no grass | 0 |
| Non habitat | None | Buildings, water bodies | -1 |

**Tabel 2. Vole habitat quality categories based on the study of Hansson (1971 & 1977).**  
  
  


---

  
  
**Design**   
  
  
***Design* *Concepts***   
  
*Emergence* - Emergent properties described by individual based state variables are: Weight. Territory size. Location (coordinates). Other emergent individual properties are: Distance moved by individuals. Realised lifespan. Individual genome. Individual location (habitat type) Reproductive output and success. Higher order emergent properties: Spatial distribution of voles. Population size and fluctuations. Sex ratios. Population reproductive output and success. Population genome.  
  
*Adaptation* - Vole movement in evaluate and explore and dispersal will provide the voles with a way to improve their basic fitness by optimising their territory quality, and maximising search range if territory quality falls. The infanticide behaviour of the male vole will improve genetic fitness.   
  
*Fitness* - Fitness is measured directly in terms of physiological reserves, size and the number of starvation days. Indirectly in the number of offspring a vole contributes to the population.   
  
*Prediction* - Prediction is not used by the vole.  
  
*Sensing* - Voles can sense the location of other voles, the age and territorial state of other voles, and their sex. They can also sense the type and structural quality of vegetation at their location. Interaction Voles interact in determining their territory locations, and breeding partners. There are also interactions between males attempting infanticide and females with young. Voles interact with predators and external events such as ploughing, harvest or mowing leading to their death. Onset of breeding is dependent upon vegetation growth, which is in turn dependent upon weather.   
  
*Stochasticity* - Stochasticity is built into the simulation at many levels. At the higher level there is the order in which voles are selected to carry out BeginStep/Step/EndStep actions. At a lower organisation level mortalities are probabilities and are implemented as a proportion e.g. the chance of ploughing mortality is 0.75 and is independently applied for each individual affected. Note however that although the chance of being killed e.g. by harvest is a probability (1 in 4), the vole will only be in a position to be subjected to this probability if it is in a field at the time the harvest operation is carried out. This is therefore deterministic in terms of the voles exposed to the causal event, but a stochastic mortality at the individual level for those so exposed.   
  
Movement also relies of probabilities of stepping from one type of habitat to another e.g. a probability of making a mistake and taking a suboptimal step, but also where two steps are equally good, then the choice is stochastic. Infanticide is based on a probability scaling with the age of the young.   
  
*Collectives* - The only collective that exists is that used to describe the young in the nest before weaning, although this does not have class status, it is a data construct of by the female.   
  
*Observation* - The PopulationManager class is the primary route for observation of the simulation. All population managers have specialised states before and after all BeginStep/Step/EndStep parts of the time-step that can be used for output. Standard ALMaSS outputs include total population size, spatially related population size via habitat type, location, farm types or vegetation types; genetic information at the individual and population level, spatial information at the population level. In addition specialised observational probes can be written to extract specific information at global or local scales (e.g. number of voles affected by pesticide toxicity). All data can be temporally referenced, and additional visual output is available in the GUI version of ALMaSS.   
  
  
For examples of vole outputs see Vole\_Population\_Manager::ImpactedProbe, Vole\_Population\_Manager::TheCIPEGridOutputProbe, Vole\_Population\_Manager::TheRipleysOutputProbe, Vole\_Population\_Manager::TheReallyBigOutputProbe  
  
***Initialisation***    
  
As far as the voles are concerned initialisation occurs by random allocation of a pre-determined number of adult male and female voles into the landscape. Voles enter the landscape with a random age between 1 & 500 days, a weight of 40g and as mature individuals with no territories. For some genetic simulations and some ecological or behavioural questions it is also possible to delimit the areas voles are started in. The landscape is initialised by running a burn-in year without the voles to allow all management plans to start off at a realistic time.  
  
**iix *Inputs***    
  
  
Input parameters vary with each application of the ALMaSS vole model but cover these basic types:   
1) Landscape structure. Implemented as a GIS map, converted to ALMaSS data structures. Input fields consist of landscape element type (e.g. field, road, hedge) and extent.   
2) Climate data on a daily basis. This data is looped if the simulation is longer than the data set. Data consists of mean daily temperature, daily precipitation and mean daily wind-speed.   
3) Values for vole model parameters see tables 1 & 2.   
4) Farming information this depends on the type and location of the farms being modelled and is implemented as a crop rotation and a set of management plans covering each crop used. These plans determine the timing and order of farm events, but these are in turn dependent on weather and farmers choices (both stochastic and mechanistic).   
5) Other values related to specific application e.g. pesticide usage, toxicity, or timing of population crashes (e.g. see Vole\_Population\_Manager::Catastrophe)  
  
  
**ix *Submodels* & Interactions**  
  
The vole model relies on the ALMaSS system for a host of services and data relating to the simulated vole environment. A key component of the vole model is the Vole\_Population\_Manager. This class is responsible for managing lists of voles in the simulation and has a number of interface functions relating to passing information to and from voles:  
  
Vole\_Population\_Manager::SupplyOlderFemales - Returns false if there is an older female within the area p\_x,p\_y +/- range.   
Vole\_Population\_Manager::SupplyHowManyVoles - Counts vole positions on the map from within a specified range of an x,y, coordinate.   
Vole\_Population\_Manager::SupplyGrowthStartDate - Returns a day in the year that the grass started to grow, if it is growing. See also Vole\_Population\_Manager::DoFirst   
Vole\_Population\_Manager::SupplyInOlderTerr - Determines if the vole is in an older voles territory.  
Vole\_Population\_Manager::SendMessage - Passes a message, currently only used for infanticide attempts.  
Vole\_Population\_Manager::SupplyVoleList - Gets a list of voles in an area.  
Vole\_Population\_Manager::InSquare - Determines if the x,y coordinate is in a given square. This is complicated by wraparound boundaries.  
Vole\_Population\_Manager::ListClosestFemales - Lists all females within p\_steps of p\_x & p\_y and returns the number in the list.   
Vole\_Population\_Manager::ListClosestMales - Lists all males within p\_steps of p\_x & p\_y and returns the number in the list.   
Vole\_Population\_Manager::FindClosestFemale - Finds the closest female.  
Vole\_Population\_Manager::FindClosestMale - Finds the closest male.  
Vole\_Population\_Manager::FindRandomMale - Finds a random male from the whole population.  
  
In addition the Vole\_Population\_Manager::DoFirst implements code that is needed before the start of a time-step. This method is called before the BeginStep of the main program loop by the base class. It controls when the grass starts to grow and therefore triggers start of reproduction but the primary role is as a safe location where outputs can be generated. At the time this is called there no chance that the voles are engaged in any kind of activity. Primary scheduling, i.e. handling of Step code and ordering of execution of vole population lists is performed by the base class Population\_Manager.  
  
Other external interactions are between all vole classes and the Landscape and Farm classes. The vole also carries genetic information which is accessed via the GeneticMaterial class. If enabled, the vole also interacts with predator objects descended from TPredator and managed by the TPredator\_Population\_Manager.  
  

---

---

Generated on Thu Jan 22 14:13:44 2009 for ALMaSS ODDox by 
 1.5.6
